# Plasma concentrations of coffee polyphenols and plasma biomarkers of diabetes risk in healthy Japanese women

**DOI:** 10.1038/nutd.2016.19

**Published:** 2016-06-06

**Authors:** A H Lee, L 'B Tan, N Hiramatsu, A Ishisaka, H Alfonso, A Tanaka, N Uemura, Y Fujiwara, R Takechi

**Affiliations:** 1 School of Public Health, Faculty of Health Sciences, Curtin University, Perth, Western Australia, Australia; 2 School of Human Science and Environment, University of Hyogo, Himeji, Japan; 3 Nutrition Clinic, Kagawa Nutrition University, Tokyo, Japan; 4 Curtin Health Innovation Research Institute, Curtin University, Perth, Western Australia, Australia

## Abstract

Coffee consumption has been reported to reduce the risk of type 2 diabetes in experimental and epidemiological studies. This anti-diabetic effect of coffee may be attributed to its high content in polyphenols especially caffeic acid and chlorogenic acid. However, the association between plasma coffee polyphenols and diabetic risks has never been investigated in the literature. In this study, fasting plasma samples were collected from 57 generally healthy females aged 38–73 (mean 52, s.d. 8) years recruited in Himeji, Japan. The concentrations of plasma coffee polyphenols were determined by liquid chromatography coupled with mass tandem spectrometer. Diabetes biomarkers in the plasma/serum samples were analysed by a commercial diagnostic laboratory. Statistical associations were assessed using Spearman's correlation coefficients. The results showed that plasma chlorogenic acid exhibited negative associations with fasting blood glucose, glycated hemoglobin and C-reactive protein, whereas plasma total coffee polyphenol and plasma caffeic acid were weakly associated with these biomarkers. Our preliminary data support previous findings that coffee polyphenols have anti-diabetic effects but further replications with large samples of both genders are recommended.

## Introduction

Coffee consumption has been reported to reduce the risk of type 2 diabetes in clinical and epidemiological studies. A recent meta-analysis of 28 prospective cohort studies concluded that the intake of caffeinated or decaffeinated coffee can reduce the diabetes risk in a dose-response manner, with one-third risk reduction by drinking six cups of coffee daily.^1^ Meanwhile, experimental studies have suggested that the polyphenols in coffee may have an important contributing role. In an animal study using wild-type mice, coffee polyphenols significantly increased gut-derived active glucagon-like peptide-1 secretion through increased intracellular cyclic AMP.^2^ Recently, an extract of coffee polyphenols was found to improve postprandial hyperglycemia and decrease oxidative stress in healthy male subjects.^3^ Nevertheless, the bioavailability and metabolism of coffee polyphenols are known to vary between individuals; as such, the plasma concentration of coffee polyphenols may more accurately reflect the ‘net exposure' of tissues and cells to these polyphenols than the estimated coffee intake.^4^ To date, there has been no report on the apparent association between coffee polyphenols in circulation and plasma biomarkers of type 2 diabetes in healthy population. Therefore, the present study aimed to determine the plasma concentrations of caffeic acid and chlorogenic acid, the two major and most abundant coffee polyphenols, in generally healthy subjects, and to assess their association with the plasma biomarkers of diabetes risk. Japanese women were investigated in view of their relatively high consumption of coffee, and coffee polyphenols provide a major source of antioxidants in the Japanese population.^5^

## Materials and methods

### Subjects

Sixty generally healthy Japanese women aged 38–73 years were recruited from Tsunashimakai Kosei Hospital and University of Hyogo in Himeji, Hyogo Prefecture of Japan, during April–August 2014. Exclusion criteria were current prescription for a chronic condition and diet modification within the past year. Informed written consent was obtained from all participants. The study protocol was approved by the Curtin University Human Research Ethics Committee (approval no. 4649) and University of Hyogo Research Ethics Committee (approval no. 068).

### Sample collection and measurement of biomarkers

Subjects were fasted overnight for more than 8 h before their blood samples being taken by a qualified phlebotomist. Plasma and serum samples were then stored at −80 °C until analysis. Anthropometric data and blood pressure were also measured before the blood sample collection. Body fat percentage was measured using a body composition scale (Tanita, Tokyo, Japan). Plasma glucose, glycated hemoglobin (HbA1c), insulin, adiponectin and C-reactive protein (CRP) were measured by a commercial diagnostic laboratory (Falco Biosystems, Hyogo, Japan).

The concentrations of caffeic acid and chlorogenic acid in plasma were determined by a highly sensitive technique using high performance liquid chromatography (HPLC; Agilent 1100LC with binary pump) (Agilent Technologies, Santa Clara, CA, USA) coupled with tandem mass spectrometer (MS/MS; Applied Biosystems Sciex API 3000, Waltham, MA, USA), as described elsewhere with minor modifications.^6, 7, 8, 9^ Briefly, 500 μl of plasma was mixed with 20 μl of 10% ascorbic acid (w/w in water) and 100 μl of 1% sulfatase H-1 (w/w in pH 5 sodium acetate buffer). After an enzymatic hydrolysis at 37 °C for 45 min, 2.5 μl of 40 μm ethyl gallate was added as an internal standard. Caffeic acid and chlorogenic acid were then extracted with 500 μl of 0.1% formic acid (v/v in ethyl acetate). The extraction process was repeated three times, and the pooled extract was evaporated under vacuum (Tomy CC-105 centrifugal concentrator, CS Bio, Menlo Park, CA, USA and Eyela Unitrap UT-2000 evaporator, Tokyo, Japan). Samples were reconstituted in 15% acetonitrile and 0.1% formic acid and then centrifuged at 15 000 *g* at 4 °C for 5 min. A total of 80 μl of the supernatant was transferred into HPLC-autosampler vials.

Five microliter of sample was next injected into Develosil ODS-SR C18, 5 μm, 2 × 150 mm column (Nomura Chemical) with binary gradient of 0.1% formic acid in ultrapure water (A) and 0.1% formic acid in acetonitrile (B) at a constant flow rate of 0.2 ml min^−1^. The gradient program began at 15% solvent B, increasing to 25% by 10 min and to 50% by 14 min, before returning to 15% which was held for 10 min. The MS/MS was operated with electrospray ionization in negative mode. The ion spray potential was −4500 V, and the source temperature was set at 450 °C.

Caffeic acid, chlorogenic acid and ethyl gallate, purchased from Sigma-Aldrich (St Louis, MO, USA), were used as a standard. The peak of each compound was identified based on a comparison of its retention times and mass spectral data with the corresponding standard and published data using the ABSCiex Analyse v1.6 software^6, 7, 8, 9^ (Supplementary Figure 1A). All samples were measured in duplicates.

### Statistical analysis

All data were managed via Microsoft Excel. ‘Total coffee polyphenol' was defined as the sum of caffeic acid and chlorogenic acid concentrations. In view of the observed non-normal distributions of the coffee polyphenol variables, the nonparametric Spearman's rank correlation method was applied to ascertain the strength of their association with the diabetes biomarkers. Statistical significance was taken at *P*<0.05.

## Results

Of the 60 healthy women recruited who met the selection criteria, 57 (95%) completed the study. Table 1 summarizes characteristics of the participants, together with their mean plasma levels of glucose, HbA1c, insulin, adiponectin and CRP, as well as their mean plasma concentrations of caffeic acid and chlorogenic acid.

Table 2 shows the Spearman's correlation coefficients between plasma coffee polyphenols and biomarkers of diabetes. Plasma chlorogenic acid was negatively associated with fasting blood glucose, HbA1c and CRP, though the strength of the association was only weak to moderate (Supplementary Figure 1B). For plasma total coffee polyphenol and caffeic acid, weak associations were found with the diabetes biomarkers. No significant association (*r*=0.10, *P=*0.44) was observed between plasma chlorogenic acid and caffeic acid concentrations (Supplementary Figure 1C).

## Discussion

Epidemiological and population studies have demonstrated promising effects of coffee consumption on reducing the diabetes risk. Such beneficial effects of coffee may be due to its high content in anti-oxidative polyphenols. Our results, based on healthy Japanese women, showed negative associations between chlorogenic acid and fasting glucose and HbA1c. Chlorogenic acid has been reported to downregulate fasting glucose and plasma glucose peak in the oral glucose tolerance test by attenuating intestinal glucose absorption.^10^ A study using murine model of diabetes revealed that chlorogenic acid can reduce fasting plasma glucose and HbA1c by modulating the adiponectin receptor signaling pathways.^11^ However, our data indicated little association between chlorogenic acid and adiponectin, and similarly for insulin, in contrast to previous reports that chlorogenic acid can increase insulin secretion.^12, 13^ It should be remarked that these experimental studies were conducted using *in vitro* islets cells of rat pancreas or glucose tolerance test in healthy subjects. Therefore, the effect of chlorogenic acid may not be apparent on fasting plasma insulin among healthy individuals.

The observed significant negative association between chlorogenic acid and CRP is consistent with the literature. In dietary-induced insulin-resistant mouse model, chlorogenic acid attenuated inflammation through decreased mRNA encoding inflammatory cytokines such as Tnfα.^14^ Furthermore, chlorogenic acid reduced TNF-α (tumor necrosis factor α), interleukin (IL)-1β and IL-6 production through attenuating toll-like receptor-mediated NF-κB signaling pathway.^15^

Interestingly, only weak associations with diabetes biomarkers were observed for caffeic acid, despite its mean plasma concentration was >30-fold greater than chlorogenic acid. Together with the low correlation between plasma caffeic acid and chlorogenic acid, our data indicated that the bioavailability and metabolism of these two coffee polyphenols may vary considerably between individuals, and chlorogenic acid appears to be more potent in reducing the diabetes risk than caffeic acid.

In conclusion, results of this study suggest that the plasma concentration of chlorogenic acid may potentially serve as an indicator of diabetes risk in healthy individuals. Further replications with large samples of both genders are recommended to confirm our preliminary findings.

## Figures and Tables

**Table 1:** Characteristics of participants, plasma coffee polyphenols and plasma biomarkers of diabetes risk.

*Characteristic*	*Means±s.d. (*n=*57)*
Age (years)	52.0±8.0
Weight (kg)	54.2±9.0
Body mass index (kg m^−2^)	22.5±4.1
Body fat percentage	24.6±6.4
Systolic blood pressure (mmHg)	116.8±12.1
Diastolic blood pressure (mmHg)	71.9±9.5
Plasma caffeic acid (nM)	54.76±40.21
Plasma chlorogenic acid (nM)	1.86±2.01
Glucose (mg dl^−1^)	94.18±36.55
Glycated hemoglobin (%)	5.44±0.96
Adiponectin (μg ml^−1^)	12.65±4.92
Insulin (μU ml^−1^)	4.82±3.13
C-reactive protein (mg dl^−1^)	0.10±0.21

**Table 2:** Association between plasma coffee polyphenols and plasma diabetes biomarkers

*Biomarkers*	*Total coffee polyphenol*	*Caffeic acid*	*Chlorogenic acid*
Glucose	0.1	0.12	−0.25*
Glycated hemoglobin	0.16	0.19	−0.25*
Adiponectin	−0.11	−0.12	0.1
Insulin	0.12	0.12	−0.01
C-reactive protein	0.02	0.02	−0.26**

Values are Spearman's correlation coefficients (*n*=57) with *P<0.1, **P<0.05.
