# Supplementary figures and images for: Plasma concentrations of coffee polyphenols and plasma biomarkers of diabetes risk in healthy Japanese women
Source: Nutr Diabetes. 2016 Jun 6;6(6):e212–. doi: 10.1038/nutd.2016.19 (PMC4931312; doi:10.1038/nutd.2016.19)

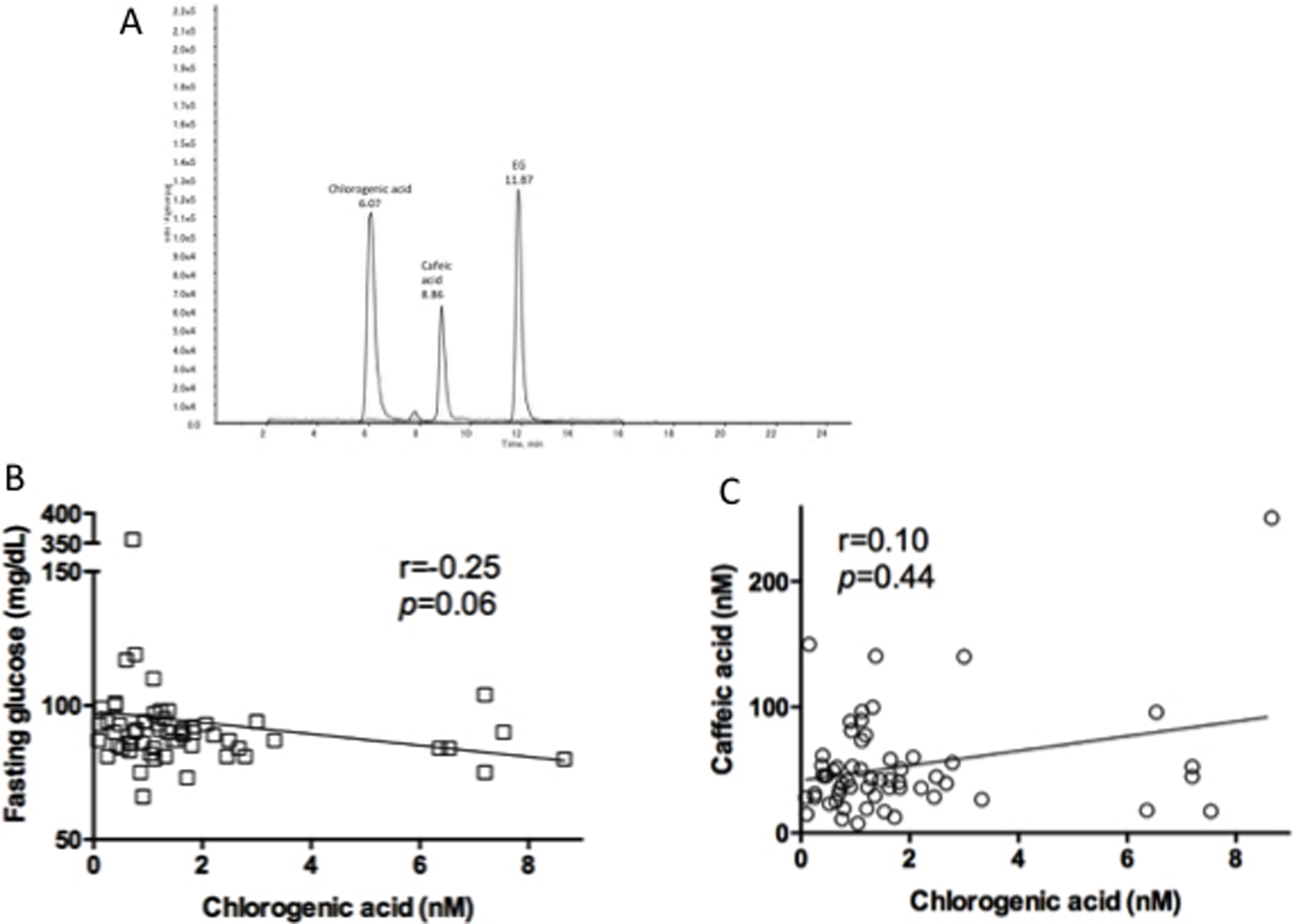

Supplement: Supplementary Figure 1 [file nutd201619x1.tif]
