# Supplementary material for: Plasma concentrations of coffee polyphenols and plasma biomarkers of diabetes risk in healthy Japanese women
Source: Nutr Diabetes. 2016 Jun 6;6(6):e212–. doi: 10.1038/nutd.2016.19 (PMC4931312; doi:10.1038/nutd.2016.19)
Supplement: Supplementary Figure Legends [file nutd201619x2.docx]

**Supplementary Figure S1. Representative HPLC-MS/MS peak chart of standards and example correlation coefficient scatter plots**

**(A)** The HPLC-MS/MS peak chart obtained from standards of chlorogenic acid and caffeic acid as well as the internal standard of ethyl gallate is shown with each corresponding retention time. Spearman’s correlation coefficient analysis was performed to investigate the association between coffee polyphenols and diabetic biomarkers (n=57). Example scatter plots between chlorogenic acid and fasting glucose **(B)**, and chlorogenic acid and caffeic acid **(C)** are shown.
